# Influence of Sex and Genetic Background on Anxiety-Related and Stress-Induced Behaviour of Prodynorphin-Deficient Mice

**DOI:** 10.1371/journal.pone.0034251

**Published:** 2012-03-29

**Authors:** Iris Kastenberger, Christian Lutsch, Herbert Herzog, Christoph Schwarzer

**Affiliations:** 1 Department of Pharmacology, Innsbruck Medical University, Innsbruck, Austria; 2 Neuroscience Program, Garvan Institute of Medical Research, Darlinghurst, Australia; Sapienza University of Rome, Italy

## Abstract

The role of dynorphin/kappa opioid receptors in epilepsy and addiction are well accepted, but their function in emotional control is not yet fully understood. Data obtained from different strains of prodynorphin (Pdyn)- and kappa opioid receptor (KOP)-deficient mice do not provide a consistent picture of the functions of Dyn/KOP in anxiety, suggesting the influence of testing conditions and/or genetic background. Therefore, we investigated the behaviour and neurochemistry of male and female Pdyn KO mice on the balb/c and C57Bl/6N background. Consistent with our results obtained from male mice on the C57bl/6N background, we observed a less anxious phenotype in the elevated plus maze, open-field and light-dark test in male mice on the balb/c background. Female mice on the balb/c background also displayed less anxiety like behaviour; however these data reflect high trait anxiety and inter-individual differences. In contrast, female mice on the C57Bl/6N background displayed low trait anxiety and a paradigm-dependent reduction of anxiety. No differences were observed in the forced swim test, while balb/c Pdyn KO mice displayed prolonged immobility in the tail suspension test. In line with our previous results, we observed reduced CRH mRNA in the central amygdala in all groups of mice. In contrast, the recently observed CRH mRNA reduction in the hypothalamic paraventricular nucleus appears restricted to male, but not female mice. Our data support previous data suggesting a pronounced impact of endogenous prodynorphin-derived peptides on anxiety. Moreover, our data support the idea that the less anxious phenotype manifests only at elevated stress levels.

## Introduction

The role of Dyn/KOP in anxiety control is not well understood. Data obtained from Pdyn- and KOP-deficient mice are relatively rare and do not provide a consistent picture of the functions of Dyn/KOP in anxiety. In Pdyn KO mice on a C57bl/6J background [1], zero-maze and startle response tests suggested an anxiogenic phenotype, while no effect was seen in the light-dark test. Femenia and colleagues [2] observed reduced time spent in the light area of the light-dark test and less time spent on the open arm of the elevated plus maze in Pdyn KO mice (C57Bl/6 non-specified), suggesting an phenotype with increased anxiety. However in both tests the number of entries appeared to be unaffected or even increased, contradicting more anxiety. In general the alterations are subtle and may be camouflaged by compensatory changes, as all models published to date are germ-line knockouts. Thus, up-regulation of both Mu and Delta opioid receptors was observed in anxiety-related brain nuclei of Pdyn and KOP KO mice [3], [4]. In our Pdyn KO mouse line, maintained on a C57bl/6N background [5], phenotype with markedly reduced anxiety was observed in three independent tests (open field, light-dark choice and elevated plus maze), which was reproduced in wild-type mice through treatment with the KOP antagonists norBNI and GNTI. The Pdyn KO phenotype was reversed by treatment with the selective KOP agonist U-50488H.

In addition, anxiety testing is strongly influenced by epigenetic and environmental conditions [6]. In line with this, Bruchas et al. [7] showed that Pdyn KO mice displayed a markedly stronger less anxious phenotype under pre-stressed compared to non-stressed conditions.

The neurochemical background of the involvement of Dyn in anxiety control appears fairly complex and KOP activation in different brain areas affects behaviour differently. Therefore, the behaviour of Pdyn KO mice hardly can be pinned down to single brain areas. However, involvement of the amygdala is very likely. Thus, reduced expression of corticotropin-releasing hormone (CRH) was observed in the hypothalamic paraventricular nucleus independently in two different strains of Pdyn KO mice [2], [5], which could be reproduced in wild-type mice by a single injection of 10 mg/kg norBNI 48 h before testing [5]. Moreover, reduced CRH expression in the central amygdala support an less anxious phenotype of Pdyn KO mice. Inhibition of synaptic transmission and LTP in the basolateral amygdaloid nucleus via activation of KOP stimulation was recently reported [8]. This nucleus plays a crucial role in anxiety control [9], and CRF1-receptor-mediated activation of the Dyn/KOP system in this nucleus was shown to cause anxiety-like behaviour in mice [7].

Similar unequivocal as the influence on anxiety-like behaviour are influences on stress-induced behaviour in Pdyn KO mice. While we observed no differences in stress-induced hyperthermia [1], reported a delayed and subtle increase in stress-induced hyperthermia in their Pdyn KO mice. This is accompanied by basal reduced corticosterone levels in our mice [5], which was not confirmed by other studies [1], [2]. Also corticosterone levels after stress appear altered. While we observed lowered corticosterone in Pdyn KO mice one hour after stress [5], Bilkei-Gorzo and colleagues [1] reported lower amplitude and delayed termination of the hormonal stress response in Pdyn KO mice. These data are not necessarily contradictive, but suggest a flatter but longer stress response in Pdyn KO mice.

Tests for stress-induced immobility (depression-like behaviour) revealed further subtle phenotypes in Pdyn KO mice. Again, these data appear unequivocal in the forced swim test. While McLaughlin et al. propose anti-depressant phenotype in Pdyn KO mice, this could not be confirmed in our study. In our hands, Pdyn KO mice displayed increased immobility at a single measurement [5], while McLaughlin et al. [10] observed reduced immobility in all measurements on the second day. Differences in immobility in the tail suspension test could be overcome by analgetic treatment and may depend on stress-induced analgesia, which was shown to be impaired in Pdyn KO mice [10].

There is vast literature on the influence of testing conditions, sex and genetic background on the outcome of behavioural testing (for review see [11]). Interindividual differences are founded as early as in-utero [12]. Maternal stress influences aggressive behaviour, potentially through co-elevation of glucocorticoids and androgens by stress [13]. Post-nataly endocrine factors play a crucial role, especially in females [14]–[16]. In males the social status influences the testosterone levels [17] and aggressive and anxiety like behaviour [18], [19]. We hypothesized that the moderate differences reported may, at least in part, depend on differences in housing and testing conditions. We further sought that a less anxious phenotype may become clearer on a genetic background with higher trait anxiety. Therefore, we backcrossed our Pdyn KO mice onto the balb/c background over eight generations and investigated their anxiety-related phenotype and neurochemistry together with female Pdyn KO mice on the C57Bl/6N background.

## Materials and Methods

### Animals

The generation of Pdyn KO mice has been described elsewhere [20]. In short, the entire coding sequence of the Pdyn gene was replaced by a basally silent construct Cre-recombinase under Teton control. Mice were backcrossed onto the balb/c and C57Bl/6N backgrounds over eight and 10 generations, respectively. For breeding and maintenance mice were group-housed with free access to food and water. Temperature was maintained at 23°C with 60% humidity and a 12 h light-dark cycle (lights on 7 am to 7 pm). Mice were tested at 3 to 6 months of age in all experiments. Age- and testing experience-matched wild-type littermates were used as controls. Tests were performed in the fixed order with fixed time intervals for all animals: open-field, elevated plus maze, light-dark choice, forced swim, and tail suspension test. All procedures involving animals were approved by the Austrian Animal Experimentation Ethics Board in compliance with the European convention for the protection of vertebrate animals used for experimental and other scientific purposes ETS no. 123. (Licence number BMWF-66.011/0018-II/10b/2009) Every effort was taken to minimize the number of animals used.

### Behavioural testing

Unless stated otherwise, mice were group-housed before testing and transferred to the ante-room of the testing facility 24–72 h before the commencement of experiments, where they were given free access to food and water. The climate and light-dark cycle remained constant. Tests were performed between 9 am and 1 pm. Test settings were in accordance with the recommendations of EMPRESS (European Mouse Phenotyping Resource of Standardised Screens; http://empress.har.mrc.ac.uk) where available. Due to the high trait anxiety of balb/c mice, the illumination in all anxiety tests was reduced to 50 lux (C57Bl/6N: 150 in the open-field, 180 in the elevated plus maze and 400 lux in the light-dark test). All tests were video monitored and evaluated by an experimenter blinded to the genotype of the animals. A fixed order of tests was kept to guarantee comparable conditions: open field, elevated-plus maze, light dark tests with one week intervals, tail suspension test and forced swim test with two weeks interval. Number of animals in different tests may vary because data of mice showing injuries (resulting from fighting), reduced whiskers or problems at the testing day (for example irregular noise outside) were rejected.

#### Open field

Open-field behaviour was tested over 10 min in a 50×50 cm open field box equipped with infrared rearing detection. Illumination was set to 150 lux for C57Bl/6N mice and reduced to 50 lux for balb/c mice according to their high trait anxiety level. This reduction resulted from previous experiments when balb/c mice didn't move at all at 150 lux and 100 lux (data not shown). The pretested animals were not included in this study. Explorative behaviour was analysed using Video-Mot 2 equipment and software (TSE-systems, Bad Homburg, Germany). Arenas were subdivided into the border (up to 8 cm from wall), centre (20×20 cm, i.e. 16% of total area), and intermediate area.

#### Elevated plus maze

Behaviour was tested over 5 min in an elevated plus maze 0.7 m above the ground, consisting of two closed and two open arms, each 50×5 cm in size. The test instrument was built from grey PVC, the height of the closed arm walls was 20 cm, while the open arms were enclosed by a rim of 3 mm. Illumination was set to 50 lux for balb/c and 180 lux for C57Bl/6N mice. Animals were placed in the centre, facing an open arm. Analysis of open and closed arm entries and time spent on the open arm was conducted automatically using Video-Mot 2 equipment and software. Entry into the open arm was recorded only when all four legs of the mouse left the neutral central area.

#### Light-dark test

Explorative behaviour in a (brightly) lit area (50 lux for balb/c; 400 lux for C57Bl/6N) was investigated by insertion of a black box into the open-field arena, covering one third of the space. One small field directly at the entrance to the black box was assigned as the transition zone. To reach the larger compartment assigned as the open area, the mouse had to completely leave the dark area. The number of entries, time spent and distance travelled in the light area were evaluated for C57Bl/6N mice. For balb/c mice entry or lack thereof into this field was recorded, as the number of entries was too low to statistically evaluate the time spent and distance travelled in this compartment in wild-type mice.

#### Tail suspension test

The tip (c.a. 1.0–1.5 cm) of the tail of the mice was securely fastened with medical adhesive tape to a metallic surface. Mice were suspended for 6 min approximately 30 cm above the surface. The illumination on the floor of the table was about 100 lux. Immobility (lasting over 2 sec) and latency to the first immobile phase of the mice was evaluated.

#### Forced swim test

This test was performed in a single 15-min trial. To increase stress we performed the test in water held at 25°C (30°C in previous experiments). Immobility, defined as no activity for at least 2 sec, was independently evaluated from video clips for the final 4 min of the test by two investigators blinded to the genotype of the animals.

### *In situ* hybridization

*In situ* hybridization was carried out as described elsewhere [5]. The following custom-synthesized (Microsynth, Balgach, Switzerland) DNA oligonucleotides complementary to mouse mRNAs were used: NPY: 5′-GAGGGTCAGTCCACACAGCCCCATTCGCTTGTTACCTAGCAT-3′; CRH: 5′-CCGATAATCTCCATCAGTTTCCTGTTGCTGTGAGCTTGCTGAGCT-3′.

To evaluate *in situ* hybridization, digitized images of the areas of interest were acquired from photo-emulsion dipped and superficially Nissl counter-stained brain slices at 200× magnification using a digital camera (Axiocam, Zeiss, Heidelberg, Germany) mounted onto a Zeiss Axiophot 2 microscope. The density of silver grains was evaluated by an experimentally blinded observer by outlining single neurons and measuring the percentage of area covered by silver grains (black grains in bright-field image, Image-J open source software available from imagej.nih.gov/ij/download/).The values of at least 30 single neurons were used to calculate the mean value for each animals. Thus, n represents the number of animals. Expression levels are given as mean percent of control. If not stated differently, brain samples were collected from testing naïve, unstressed animals.

### Serum analyses

Testing naïve, unstressed animals were killed by decapitation between 12.00 and 14.00 hours while under deep CO_2_ anaesthesia. Trunk blood was captured and serum was stored at −20°C until analysis. Corticosterone serum levels were measured using a commercial radioimmunoassay (MP Biochemicals, Orangeburg, NY) according to the manufacturer's instructions. Each serum was analysed in duplicate.

### Statistical analysis

The Student's t test was used for all comparisons of the two genotypes, with the exception of the analysis of the behaviour of male balb/c mice in the light-dark test. Here we applied a Chi^2^ test to the distribution frequency of classification data. Comparison of more than two groups was carried out by One-Way-ANOVA, followed by Bonferroni's multiple comparison test, using GraphPad Prism 5.0 software. P-values of <0.05 were accepted as statistically significant. All data are given as mean ± SEM (n).

## Results

### Male balb/c mice

#### Open-field test

**Figure 1:**
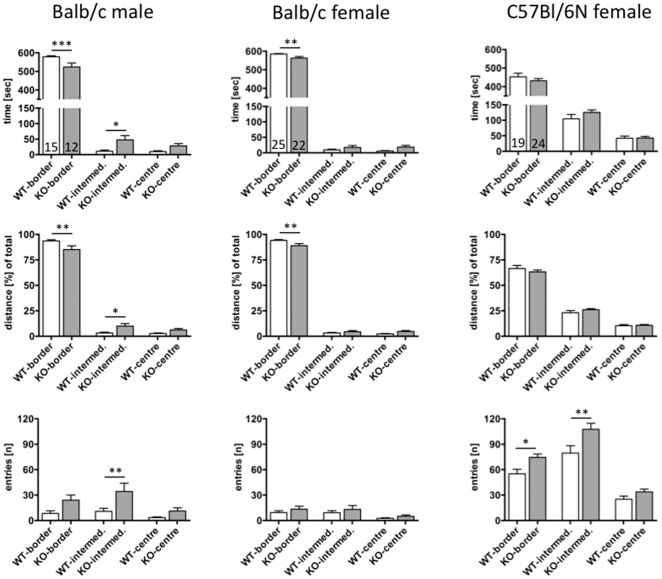
Ambulation in the open field by Pdyn KO (shaded bars) and WT (white bars) mice was measured for 10 min. Time spent (upper row), distance travelled (mid row) and number of entries (lower row) into the different zones are given for male (left column) and female (mid column) balb/c and female C57Bl/6N (right column) mice. Data represent mean ± SD of the number of animals indicated in the bars. Statistical analysis was performed by one-way ANOVA with Bonferroni's multiple comparison test for comparison of the two genotypes in the different zones of the open-field test. * … p<0.05; ** … p<0.01; *** … p<0.001.

Pdyn KO mice spent significantly more time (intermediate 11±3.9 sec (15) vs. 47.7±13.8 sec (12); p = 0.0001; centre 9.7±3.0 sec (15) vs. 28.4±7.3 sec (12); p = 0.0084; WT vs. Pdyn KO, respectively) and travelled more distances (intermediate 3.2±0.8% (15) vs. 10.0±2.5% (12); p = 0.0014; centre 2.7±0.5% (15) vs. 6.2±1.4% (12); p = 0.0049; WT vs. Pdyn KO, respectively) in the intermediate and central areas of the open field than WT mice. In addition, the number of entries into these areas was higher in Pdyn KO mice (intermediate 10.8±3.6 (15) vs. 34.2±9.7 (12); p = 0.0029; centre 3.3±0.9 (15) vs. 11.2±3.8 (12); P<0.0001; WT vs. Pdyn KO, respectively). Due to high inter-individual differences statistical significance was only reached for the border and intermediate areas, and not for the centre (Fig. 1A).

#### Elevated plus maze

**Figure 2:**
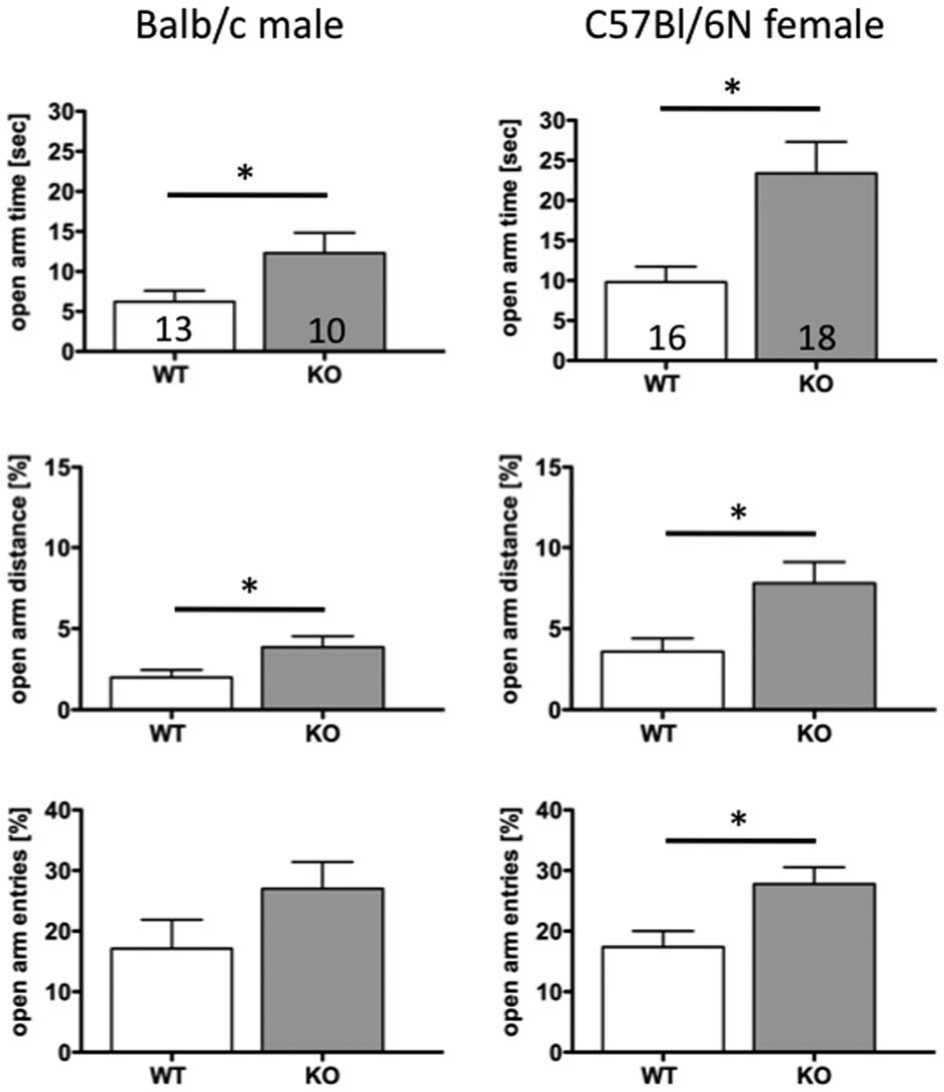
Ambulation on the elevated plus maze by Pdyn KO (shaded bars) and WT (white bars) mice was measured for 5 min. Time spent (upper row), distance travelled (mid row) and percentage of entries (lower row) onto the open arms are given for male balb/c (left column) and female C57Bl/6N (right column) mice. Data represent mean ± SD of the number of animals indicated in the bars. Student's t test was used to compare the two genotypes. * … p<0.05.

Pdyn KO mice displayed more exploratory behaviour on the open arm than WT mice (Fig. 2). This is reflected by the significantly longer distance travelled (1.98±0.47% (n = 13) vs. 3.86±0.67% (n = 10); p = 0.0277 WT vs. Pdyn KO, respectively) and time spent on the open arms (6.2±1.38 sec. (n = 13) vs. 12.3±2.53 sec. (n = 10); p = 0.0353; WT vs. Pdyn KO, respectively). The number of visits to both the closed (33.5±4.83 (n = 13) vs. 29.7±6.14 (n = 10); p = 0.6264; WT vs. Pdyn KO, respectively) and open arms (7.7±2.14 (n = 13) vs. 9.7±1.46 (n = 10); p = 0.4879; WT vs. Pdyn KO, respectively) did not appear to differ (Fig. 2).

#### Light-dark choice test

Balb/c mice are known to be very shy in the light-dark test. In fact, balb/c WT mice very rarely exit the dark compartment. Therefore, the usual evaluation of entries, time and distance in the light area could not be applied. However, we still observed a significantly higher number of animals entering the light area in the Pdyn KO group (9 out of 15) as compared to WT (1 out of 20) animals (p = 0.0004; Chi^2^ test).

#### Forced swim test

The forced swim test was performed in a single 15-min session. No marked differences in immobility were observed over the last 4 min of this test (68.1±15.7 sec. (n = 10) vs. 71.7±23.6 sec. (n = 7); p = 0.8964 WT vs. Pdyn KO, respectively).

#### Tail suspension test

Statistically significant differences were observed for both the time spent immobile and the latency to the first immobile phase between WT and Pdyn KO mice. While WT mice spent 15.8±6.0 sec (n = 9) immobile, Pdyn KO mice were immobile for an average of 56.4±10.8 sec (n = 7) (p = 0.0038). The onset of the first immobile period was observed in WT mice after 239±23.6 sec (n = 9), while Pdyn KO mice displayed the first immobile phase after 149±117 sec (n = 7; p = 0.1411). One of the WT, but none of the Pdyn KO mice climbed along their tail to the bar and had to be removed from evaluation.

#### Neurochemistry

*In situ* hybridization revealed a reduction of CRH mRNA in the central amygdala (100±11.5 (n = 4) vs. 78±8.7 (n = 5); p = 0.0154; WT vs. Pdyn KO, respectively) and in the paraventricular nucleus (100±8.2 (n = 5) vs. 82±8.8 (n = 5); p = 0.0088; WT vs. Pdyn KO, respectively) of balb/c Pdyn KO mice. NPY mRNA was increased in neurons of the basolateral amygdala of Pdyn KO mice (100±6.5 (n = 5) vs. 117±3.3 (n = 5); p = 0.0008; WT vs. Pdyn KO, respectively), but not in the central amygdaloid nucleus (100±3.0 (n = 4) vs. 99±5.1 (n = 5); p = 0.7406; WT vs. Pdyn KO, respectively). Data represent the mean percent of control ± SD (n).

#### Corticosterone serum levels

No marked differences in serum corticosterone levels were observed between Pdyn KO and WT mice (73.3±34.4 ng cort./ml (n = 11) vs. 52.0±23.3 ng cort./ml (n = 11); p = 0.1046; WT vs. Pdyn KO, respectively).

### Female mice

Female mice on C57Bl/6N and balb/c backgrounds were tested. However, valid data for all tests were only obtained from the C57Bl/6N mice. Balb/c mice were very shy, even at reduced light intensities, and therefore allowed only limited evaluation.

#### Open-field test

Female mice on the balb/c background rarely left the border zone, and therefore statistical analysis of ambulation in the intermediate and central zones did not reveal significant differences. On the other hand, Pdyn KO mice spent less time (588±2.21 sec (n = 12) vs. 546±17.4 sec (n = 8); p = 0.0102; WT vs. Pdyn KO, respectively) and travelled a shorter distance (95±2.79% (n = 12) vs. 87±3.07% (n = 8); p = 0.0019; WT vs. Pdyn KO, respectively) in the border zone, reflecting increased exploration of non-border areas (i.e. intermediate+central zone) regarding time (9.0±1.92 sec (n = 12) vs. 49.4±16.7 sec (n = 8); p = 0.0065 and distance travelled (4.9±0.81% (n = 12) vs. 13.1±3.07% (n = 8); p = 0.0065; WT vs. Pdyn KO, respectively; Fig. 1B). Female Pdyn KO mice on the C57Bl/6N background did not behave markedly differently from WT mice. However, the number of transitions between the border and intermediate area increased significantly by 35% (Fig. 1C).

#### Elevated plus maze

Female Pdyn KO mice on the C57Bl/6N background displayed significantly increased ambulation on the open arms compared to WT mice (Fig. 2). This was reflected by increased time (9.8±1.93 sec (n = 16) vs. 23.4±3.91 sec (n = 18); p = 0.0051; WT vs. Pdyn KO, respectively), increased distance (3.4±0.79 m (n = 16) vs. 9.6±1.58 m (n = 18); p = 0.0019; WT vs. Pdyn KO, respectively) and more entries (2.6±0.43 (n = 16) vs. 5.4±0.86 (n = 18); p = 0.0079; WT vs. Pdyn KO, respectively) onto the open arms (Fig. 2). Entries into closed arms did not appear to differ (11.5±1.22 (n = 16) vs. 13.9±1.75 (n = 18); p = 0.2805; WT vs. Pdyn KO, respectively). Balb/c mice rarely entered the open arms even in low light conditions, and therefore we evaluated the number of pokings into the open arm. This was done by introducing a small field (5×5 cm) at the entry of the open arms. To enter this field, mice did not need to completely leave the neutral central zone and therefore it was less aversive than the open arms themselves. Pdyn KO mice explored this “poking area” more frequently (5.4±1.09 entries (n = 14) vs. 22.1±9.06 entries (n = 8); p = 0.0245; WT vs. Pdyn KO, respectively) and for longer (3.7±1.02 sec (n = 14) vs. 19.3±9.03 sec (n = 8); p = 0.0332; WT vs. Pdyn KO, respectively) than wild-type mice. We also looked at the number of pokings of C57Bl/6N female mice, but no differences were found, showing that C57Bl/6N entered the full open arm when leaving the neutral zone of the elvated plus maze.

#### Light-dark choice test

Statistically significant differences between WT and PdynKO mice on the C57Bl/6N background were observed for the number of entries into the light area (3.4±0.52 (n = 16) vs. 6.1±1.14 (n = 18); p = 0.0473; WT vs. Pdyn KO, respectively), but not for the distance travelled in this area (298±42.2 cm (n = 16) vs. 344±67.2 cm (n = 18); p = 0.5773; WT vs. Pdyn KO, respectively). Only 4 PdynKO mice and none of the WT out of 22 mice on the balb/c background entered the light area, and therefore, this test could not be evaluated.

#### Forced swim test

The forced swim test was performed in a single 15-min session. No marked differences in immobility were observed over the last 4 min of this test between WT and Pdyn KO mice on the C57Bl/6N (89±49.8 sec (n = 17) vs. 78±44.2 sec (n = 19); p = 0.4952; WT vs. Pdyn KO, respectively) or on the balb/c (91±31.8 sec (n = 11) vs. 94±43.0 sec (n = 15); p = 0.8319; WT vs. Pdyn KO, respectively) background.

#### Tail suspension test

Pdyn KO mice on the balb/c background spent a prolonged time immobile (43.2±28.9 sec (n = 10) vs 74.5±40.8 sec (n = 15); p = 0.0474; WT and Pdyn KO respecitively), while the delay to to onset of the first immobile phase did not reach statistical significance (127±60,4 sec (n = 10) vs 88±60.7 sec (n = 15); p = 0.1266; WT and Pdyn KO respecitively). No statistically significant differences were observed for either the time spent immobile or the latency to the first immobile phase between WT and Pdyn KO mice on the C57Bl/6N background. While WT mice spent 73±49.9 sec (n = 22) immobile, Pdyn KO mice were immobile for an average of 74±43.1 sec (n = 32) (p = 0.8954). The onset of the first immobile period was observed in WT mice after 81±39.6 sec (n = 22), while in Pdyn KO mice the first immobile phase occurred after 78±33.7 sec (n = 32; p = 0.7240).

#### Neurochemistry

*In situ* hybridization revealed a reduction in the levels of CRH mRNA in the central amygdala (100±5.8% (n = 10) vs. 74±3.8% of control (n = 10); p = 0.0014; WT vs. Pdyn KO, respectively) but not in the paraventricular nucleus (100±3.2% (n = 10) vs. 92±4.2% of control (n = 10); p = 0.1472; WT vs. Pdyn KO, respectively) of C57Bl/6N Pdyn KO mice. Similar results were obtained from Balb/c mice (CeA: 100±2.6% (n = 6) vs. 85±1.35% of control (n = 4); p = 0.0029; WT vs. Pdyn KO, respectively; PVN: 100±4.7% (n = 6) vs. 96±5.0% of control (n = 4); p = 0.5874; WT vs. Pdyn KO, respectively). Levels of NPY mRNA were unchanged in neurons of the basolateral amygdala of female mice on both backgrounds (97±5.0% (n = 10) and 93±3.1% of control (n = 6); C57Bl/6N and Balb/c, respectively).

## Discussion

Investigation of the involvement of dynorphin in anxiety control has produced conflicting results to date. The phenotypes observed in different Pdyn KO mouse lines range from markedly anxiolytic [5] over paradigm-dependent anxiolytic [1], [7] to anxiogenic [2]. These studies used different strains of Pdyn KO mice, on partially different genetic backgrounds, and applied different setups for behavioural testing. To investigate the influence of sex and genetic background, we investigated female C57Bl/6N and both sexes of balb/c Pdyn KO mice under identical housing conditions and testing arenas as used previously [5].

Our present data confirm an anxiogenic role for prodynorphin-derived peptides. Thus balb/c Pdyn KO mice displayed increased ambulation in all three anxiety-related tests, backing up the data obtained for mice on the c57bl/6N background [5]. However, the high trait anxiety level of balb/c mice was reflected in the results of a number of our tests. Thus, the increase in ambulation in aversive areas in the open-field test was significant only in the intermediate zone, which is less aversive than the centre. Evaluation of the elevated plus maze and light-dark choice test was also adapted to this high trait anxiety. Wherever evaluation was feasible, Pdyn KO mice were statistically significantly less anxious than wild-type mice.

In contrast to balb/c mice of both sexes and male C57Bl/6N mice, female Pdyn KO C57Bl/6N mice displayed reduced anxiety in dependence of the testing paradigm. Increased ambulation in aversive zones in the elevated plus maze was opposed by unchanged behaviour in the open-field or light-dark choice tests. This may be due to the lower overall anxiety observed in female rodents [21], [22], as the open-field test and the light-dark test are seen as less challenging than the elevated plus maze. This would be also consistent with the proposal of Bruchas et al. [7], who proposed that the less anxious phenotype of Pdyn KO mice is only detectable under highly challenging conditions.

In a recent study [5], we suggested that decreased expression of CRH in the central amygdala might represent an important feature of the less anxious phenotype of Pdyn KO mice. This alteration in neurochemistry was observed in all mice strains and sexes investigated, suggesting that down-regulation of CRH expression in the central amygdala is actually induced by the lack of Pdyn. Noteworthy, it was suggested that Dyn is released in response to stress [7], [23]. This is associated with increased phosphorylation of KOP in the basolateral amygdala after CRF injection [7]. Interestingly, this could be blocked by CRF_2_ receptor antagonists [23], but was also absent in CRF_1_ receptor deficient mice [7]. Thus, it was concluded that CRF induced Dyn release may induce different pathways, which both are KOP dependent. Indeed, behavioural experiments suggest that CRF1 receptor mediated Dyn release is responsible for aversive effects [7], while CRF2 receptor mediated Dyn release produced rewarding effects in conditioned place preference tests [23]. Our data support a close interrelation of CRF and Dyn, however suggest that this does not follow a one-way direction.

In line with our recent results obtained from C57Bl/6N mice, male Pdyn KO balb/c mice also displayed longer lasting and earlier immobility than wild-type mice in the tail suspension test. This phenotype appeared less pronounced in female balb/c mice and was not observed in female Pdyn KO C57Bl/6N. In contrast, no marked differences in behaviour were observed in the forced swim test, regardless of genetic background or sex. However there are contrary findings regarding activation of KOP and the behavioural response to stress. Thus, McLaughlin et al. reported disrupted stress response from Pdyn deficient mice [10], while prolonged stress response was suggested by Bilkei-Gorzo et al. [24]. It has been suggested that endogenous dynorphins play a role in stress-induced analgesia [10], [25]. In fact, differences in immobility between the genotypes were abolished by analgesic treatment in our recent study [5]. The sex differences in the tail suspension test may at least in part reflect the increased stress resistance of female mice. It is noteworthy that potential influences of Dyn on the hypothalamic-pituitary-adrenal axis, as suggested by the reduction in CRH expression in the hypothalamic paraventricular nucleus in male Pdyn KO mice [5], were confirmed in male balb/c but not female mice in the present study. However, decreased basal corticosterone serum levels did not reach statistical significance either in male or female balb/c or female C57Bl/6N mice. This is consistent with the study of Bilkei-Gorzo et al., who reported alterations in stress response duration, but not basal corticosterone serum levels in their Pdyn KO mice [1].

### Conclusions

Based on the behavioural and neurochemical data of our recent and present studies and the data obtained by other groups, we suggest that the influence of dynorphin on anxiety control predominantly depends on the relationship between the trait anxiety and stress resistance of mice and the level of aversion in the test setup. Thus, Pdyn KO on the balb/c background with high trait anxiety displayed anxiolytic-like phenotypes in all tests and both sexes. In contrast, Pdyn KO on the less anxious C57Bl/6N background do not. Female mice (which display lower overall anxiety than males) only appeared less anxious in the elevated plus maze test.

It is noteworthy that male Pdyn KO on this background only displayed increased entry into the light area of the light-dark choice test at 400, and not at 150 lux [5]. In line with this, Bruchas et al. [7] reported that Pdyn KO mice displayed a clear less anxious phenotype in the elevated plus maze at high, but not low, illumination levels. Under low brightness (40 lux) and in a small open-field arena (25×25 cm), male Pdyn KO mice on the C57Bl/6 background showed no anxiolytic-like behaviour [2].
